# Long-term functional outcome and quality of life following rotationplasty for treatment of malignant tumors

**DOI:** 10.1186/s12891-015-0721-0

**Published:** 2015-09-24

**Authors:** Guntmar Gradl, Lukas K. Postl, Ulrich Lenze, Josef Stolberg-Stolberg, Florian Pohlig, Hans Rechl, Markus Schmitt-Sody, Ruediger von Eisenhart-Rothe, Chlodwig Kirchhoff

**Affiliations:** Department of Orthopedics and Sports Orthopedics, Klinikum rechts der Isar, Technische Universitaet Muenchen, Ismaningerstrasse 22, 81675 Munich, Germany; Department of Trauma Surgery, Klinikum rechts der Isar, Technische Universitaet Muenchen, Munich, Germany; Department of Orthopedics, Klinikum der Universitaet Muenchen, Ludwig-Maximilians-Universitaet, Munich, Germany

**Keywords:** Rotationplasty, Bone tumor, Sarcoma, Quality of life, Function, Long-term

## Abstract

**Background:**

Malignant bone tumors of the lower extremity are more frequently found in children and adolescents than in adults. Modern treatment regimens led to high limb salvage rates and offer the choice between endoprosthetic replacement and rotationplasty in many cases. Rotationplasty has proven to be an effective, highly functional option in short- and mid-term studies. Aim of this study was to assess long-term results regarding quality of life and functionality after rotationplasty and to compare the obtained results to a representative healthy German sample cohort.

**Methods:**

In total 12 patients who underwent rotationplasty between 1991 and 2001 were enrolled in this study. After physical examination, they were evaluated regarding health related quality of life, functional outcome and psychosocial status. While quality of life was mainly assessed using the SF-36 (The Short Form (36) Health Survey v2), functional outcome was measured using the musculoskeletal tumor society score (MSTS) as well as the Tegner activity level scale.

**Results:**

Average age at the time of surgery was 19 ± 10 year. and 32 ± 11 year. at the time of follow up. Mean follow-up was 14 ± 9 years. The SF-36 scores accounted for 80.4 ± 15.7 regarding physical functioning, for 78.1 ± 24.1 regarding the physical role functioning, for 74.1 ± 17.6 regarding bodily pain and for 71.8 ± 26.1 regarding general health. SF-36 score for vitality was 75.0 ± 12.8, for social functioning 98.9 ± 3.6, 88.2 ± 23.9 for emotional role functioning and 89.6 ± 10.1 for the mental health. Comparison to a representative German sample cohort revealed significantly higher patient’s scores for vitality, social functioning and mental health (p < 0.05). The overall MSTS resulted in an average of 64 ± 12 % and the Tegner activity level scale accounted for 4.1 ± 0.6 pts.

**Conclusions:**

The presented long-term results indicate that rotationplasty provides a high quality of life. Patients are satisfied with a good functional outcome regarding activities of daily life and even sports.

## Background

Sarcomas account for approximately 1 % of all adult cancers [1]. In this context soft tissue sarcomas typically occur in middle aged and older adults [1], whereas malignant bone tumors, *i.e.,* osteosarcoma and Ewing’s sarcoma are more frequent in children and adolescents [2, 3]. Progress in modern treatment regimens including neoadjuvant and adjuvant therapy has markedly improved the overall survival rates over the last decades [4]. Therefore, analysis of long-term outcome after malignant bone tumor therapy gains more and more importance. Moreover only a minor percentage of patients require primary amputation due to the efforts of limb preserving surgical procedures [3, 5–9]. Nowadays endoprosthetic knee replacement is associated with good functional, cosmetic and psychological outcomes, resulting in significantly better walking efficiency and musculoskeletal tumor society scores (MSTS) in comparison to major amputation or arthrodesis [10–12].

In contrast rotationplasty has been proven to be associated with equivalent functional outcomes, but better quality of life as well as less limitations during daily activities and less pain in the short- and mid-term outcome [13–17]. However, especially the cosmetic result is not as appealing as for traditional limb sparing alternatives. In this context only a small number of studies investigated the health-related quality of life (HRQL) and the long-term outcome after rotationplasty for the treatment of malignant bone tumors [18–20]. Therefore the aim of this study was to assess long-term results following rotationplasty with regard to HRQL, functional performance and psychosocial aspects and to compare theses results to a representative healthy German sample cohort [21].

## Methods

Ethical approval for this project was granted by the local ethics committee (Klinikum rechts der Isar, Medical Faculty, reference no. 4092/11). Written informed consent was obtained from each patient prior to enrolment in the study.

### Patients

All patients who had been treated by rotationplasty for malignant bone or soft tissue tumors of the lower extremity at our academic musculoskeletal tumor center (MSTC) between May 1991 and June 2001 were identified from our database.

### Questionnaires

Before assessing the questionnaires all patients were clinically examined by an expert orthopedic surgeon (GG).

The Short Form Health Survey (SF-36v2) was used for the assessment of HRQL [22]. The SF-36v2 is a questionnaire assessing the individual health of patients as well as disease-related distress by eight scaled dimensions. Each scale ranges from zero (poor) to 100 (excellent). The eight dimensions are *vitality, physical functioning, bodily pain, general health perceptions, physical role functioning, emotional role functioning, social role functioning* and *mental health* [22].

The functional outcome was evaluated using the musculoskeletal tumor society score (MSTS) [23]. The MSTS evaluates the functional outcome of tumor patients after completed therapy. It consists of six components for the lower limb: pain, function, emotional acceptance, need for walking aids, walking and gait. The maximum (best) score for each item is 5 (range from 0 – 5). The values of each of the six components are added and divided by the maximum possible number of pts. (30). The percentage value is obtained by multiplying the calculated point value by 100.

In addition the Tegner activity level scale [24] was assessed. The Tegner activity level scale ranges from zero to ten. While an activity level of zero means sick leave or disability because of knee problems, a level of five means that the patients are able to perform heavy labor and recreational sports twice a week and a level of ten means that the person pertains to the national elite in competitive sports [25].

### Statistics

Statistical analysis was performed using Sigma Stat 3.1 software (Systat Inc, Chicago, Illinois, USA. Unless otherwise stated the data is given in means (arithmetic mean) ± standard deviation (SD). Welch’s *t*-test (two-sample unpooled *t*-test for unequal variances) was calculated for comparing SF-36v2 results of our patients to a representative healthy German sample cohort [21] using QuickCalcs software (GraphPad Software Inc, La Jolla, California, USA). A p-value < 0.05 was considered statistically significant.

## Results

### Patients

Overall 23 patients underwent rotationplasty between 1991 and 2001. Nine patients deceased due to the malignant disease, two patients were lost for follow-up. Summarizing, twelve patients (7 male, 5 female) with a median age at the time of treatment of 19 ± 10 year. and a median age of 33 ± 11 year. at follow up were enrolled. The mean follow-up was 14 ± 9 years. The diagnoses consisted of osteosarcoma (*n* = 9), chondrosarcoma (*n* = 2) and one synovial sarcoma (for patient’s details see Table 1). Patients, suffering from osteosarcoma were treated according to the Cooperative Osteosarcoma Study Group (COSS) protocol and received multidrug chemotherapy before and after surgery [26]. The patient suffering from synovial sarcoma underwent additional neoadjuvant and adjuvant chemotherapy following the consensus of our interdisciplinary tumor board.

**Table 1:** Overview of patient characteristics including diagnosis, treatment, complications and postoperative social status (*n* = 12)

Gender	
male	7
female	5
Age at surgery (yrs of age)	
Mean	19 ± 10
Median (Range)	18 (4–46)
Follow up (yrs)	
Mean	14 ± 3
Median (Range)	15 (8–18)
Diagnosis	
Osteosarcoma	9
Chondrosarcoma	2
Synovial sarcoma	1
Anatomic Site	
Distal Femur	9
Proximal Femur	3
Treatment	
Borggreve Rotationplasty	10
Winkelmann Rotationplasty	2
Winkelmann Classification	
A I	8
B I	1
B II	2
B IIIa	1
Complications	
Implant loosening	1
Impingement	1
Achilles tendon tear	1
Marital state	
Married	5
Divorced	-
Never Married	7
Living together with Partner	1
Living separated from Partner	1
Single living with parents	1
Single living alone	4
Education	
Less than compulsory	1
Compulsory	8
Postcompulsory	3
University level	-
Employment state	
Full-time job	7
Part-time job	1
Student	3
Illness retirement	1

Referring to the classification according to Winkelmann et al. in eight cases an AI type, in one case a BI, in two cases a BII and in another case a BIIIa-rotationplasty was performed [27].

### Complications

One patient underwent one revision surgery once due to prolonged healing of the osteotomy and implant loosening. Another patient had to be treated for impingement of the Borggreve joint. One patient needed ten revision procedures due to traumatic Achilles tendon tear with consecutive postoperative wound infection. There were no further major complications recorded.

### Health-Related Quality of Life (HRQL)

Analysis of the psychosocial outcome, measured by the SFv2-36 revealed for the subcategory physical health 80.4 ± 15.7 for the dimension *physical functioning*, 78.1 ± 24.1 for *physical role functioning*, 74.1 ± 17.6 for *bodily pain* and 71.8 ± 26.1 for *general health*.

Scores for the subcategory mental health state were 75.0 ± 12.8 for *vitality*, 98.9 ± 3.6 for *social functioning*, 88.2 ± 23.9 for *emotional role functioning* and 89.6 ± 10.1 for *mental health* (for details see Table 2). The comparison of the presented results to a representative healthy German sample cohort (mean age 49 ± 18 years) revealed significantly higher scores for the patients regarding the dimensions *vitality* (*p* = 0.0243), *social functioning* (*p* = 0.0001) and *mental health* (*p* = 0.0001) [21].

**Table 2:** Results of the SFv2-36 questionnaire assessing function and mental health of patients after rotationplasty of our series and a representative German sample [21]

Subjects	Physical functioning	Physical Role	Bodily pain	General health	Vitality	Social role	Emotional role	Mental health
Rotationplasty (*n* = 12)	80.4 ± 15.7	78.1 ± 24.1	74.1 ± 17.6	71.8 ± 26.7	75.0 ± 12.8	98.9 ± 3.6	88.2 ± 23.9	89.6 ± 10.1
Representative healthy German sample cohort (*n* = 2043) [21]	87.2 ± 20.4	81.8 ± 23.1	79.3 ± 25.3	64.4 ± 15.2	65.3 ± 18.3	87.5 ± 19.3	84.7 ± 22.7	72.3 ± 17.2
*p*	0.1635	0.6064	0.3309	0.3581	0.0243	0.0001	0.6229	0.0001

### Functional outcomes

**Fig. 1:**
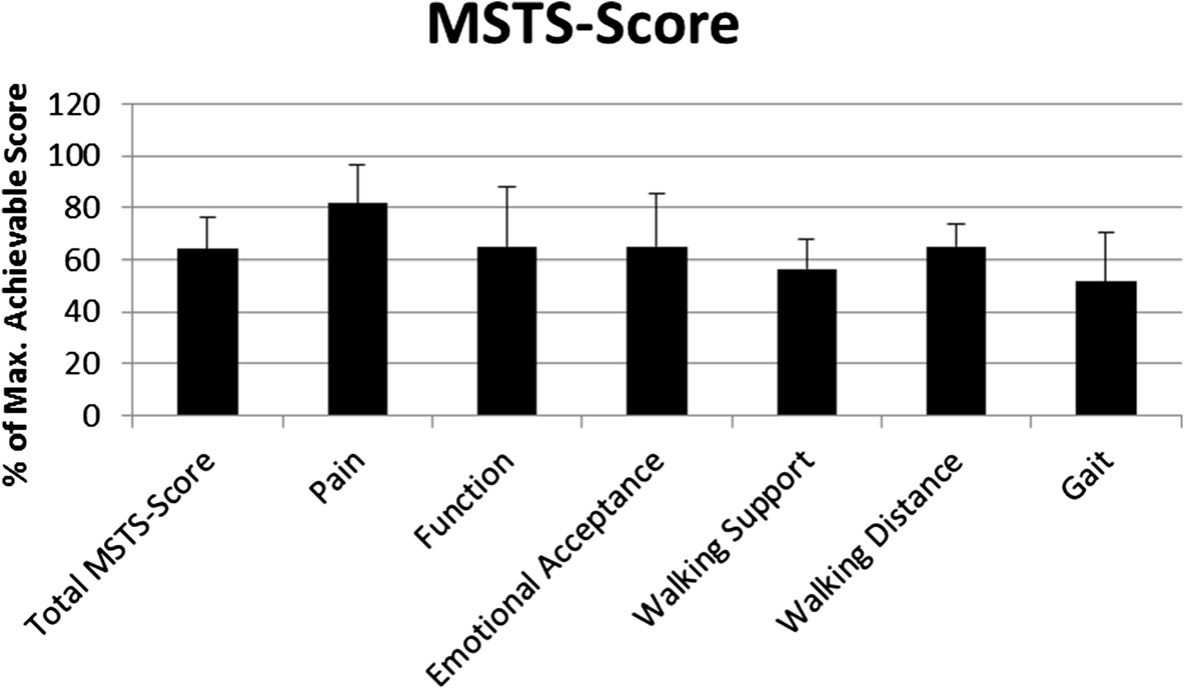
Percentage of maximum MSTS-Score and sub-scores of patients after rotationplasty

**Fig. 2:**
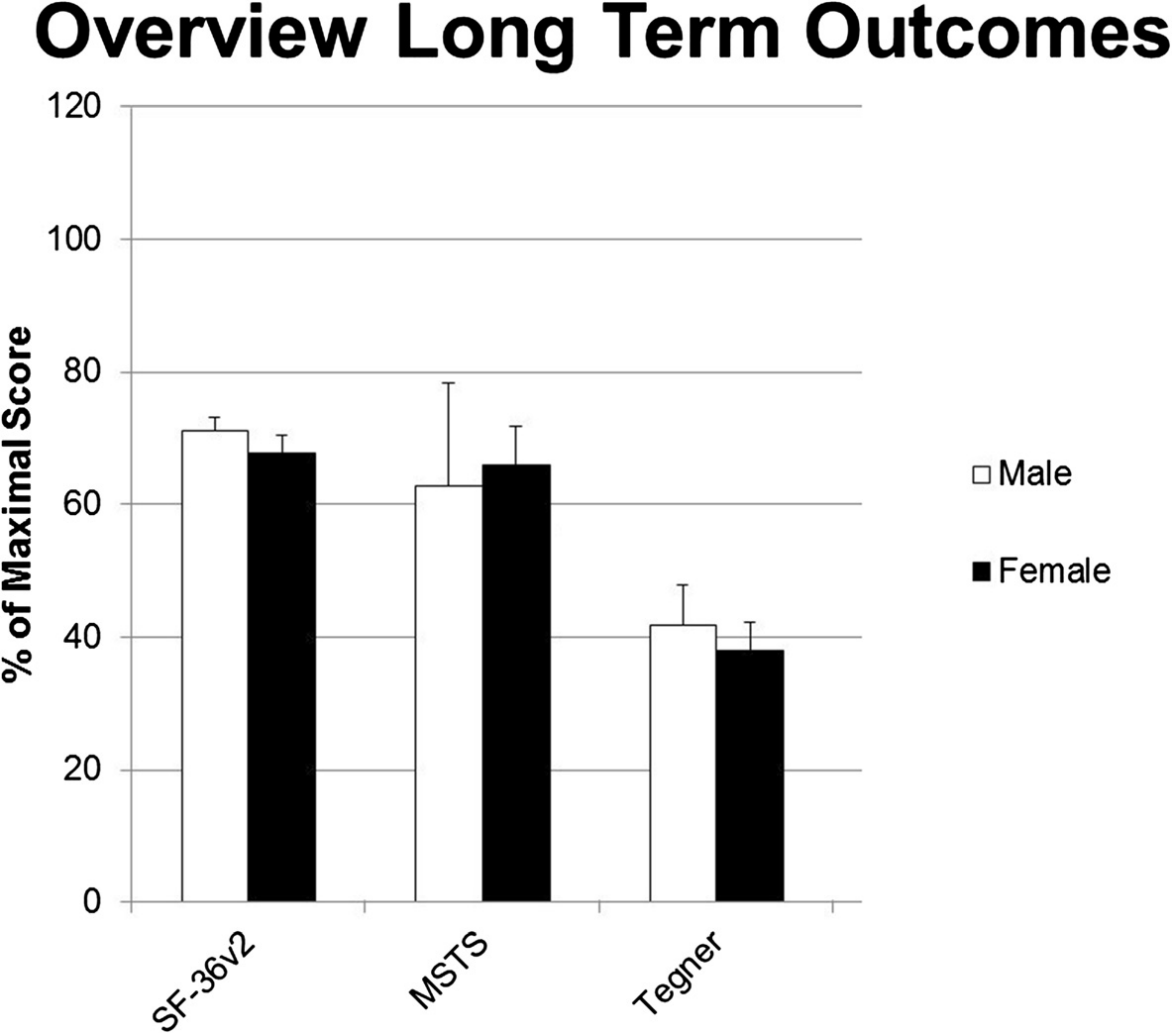
Overview of percentages of maximum score of the assessed questionnaires with standard deviations. White bars for male and black bars for female patients

The overall MSTS resulted in an average of 64 ± 12 % with 63 ± 15.4 % for male and 66 ± 5.7 % for female patients. Analysis of the subcategories revealed 4 ± 0.8 pts. for pain, 3.3 ± 1.2 for function, 3.3 ± 1 for emotional acceptance, 2.8 ± 0.6 for walking supports, 3.3 ± 0.4 for walking distance and 2.6 ± 1 for gait (see Fig. 1). The Tegner activity level scale accounted for 4.1 ± 0.6 pts (see Fig. 2).

Ten patients reported no or occasional pain, two complained about moderate daily pain. Only five patients reported limitations during recreational activities and one patient was limited during daily activities. The walking distance of nine patients was greater than 6 blocks and within the range of 4–6 blocks for the remaining three patients. Five patients had no and seven patients some difficulties while walking on uneven terrain. In seven patients the gait pattern was not or slightly and in five patients obviously altered.

Ten patients reported, having little problems in participating in sports, one of the patients is a national champion in handicapped swimming and the one patient with Achilles tendon tear performs no sports at all.

Regarding range of motion of the neo-knee flexion and extension corresponded to the levels of the contralateral ankle joint with a mild restriction (10°) in eleven patients and a moderate restriction 15° in one patient. In- and eversion was normal in all patients. The joint was regarded stable (varus/valgus stability) in all patients.

Four patients even had children following the rotationplasty. Only one patient is currently not able to work because his work as storekeeper requires carrying heavy loads. All but one patient, who was not sure, would choose a rotationplasty as treatment option again, facing the choice between amputation, endoprosthetic knee replacement or rotationplasty.

## Discussion

The objective of this retrospective study was to evaluate the long-term outcome as well as health-related quality of life (HRQL) in patients who underwent rotationplasty for treatment of a malignant bone or soft tissue tumour. At a mean follow-up of 14 ± 9 years. HRQL, assessed by the Short Form Health Survey (SF-36v2) revealed good to excellent scores for each dimension. Interestingly the scores for vitality, social functioning and mental health were significantly higher compared to a representative healthy German sample cohort [21]. Functional long-term outcome measured by the musculoskeletal tumor society score (MSTS) and the Tegner activity level scale also revealed a good functional outcome with 64 ± 12 % and 4.1 ± 0.6 pts., respectively. All but one patient, who was not sure, would choose rotationplasty as therapy option again.

Rotationplasty implies significant shortening of the leg and rotation of the foot 180° around the vertical axis [28]. Therefore the former ankle adopts the role of a neo-knee with ankle dorsiflexion simulating knee flexion [29, 30]. Although the procedure allows for a function more akin to transtibial amputation due to retention of voluntary control of motion at the ‘knee’ level the significant cosmetic alteration implies potential future socio-psychiatric issues that might impact health and HRQL [31]. However, in our cohort we found good to excellent results regarding the subcategory physical health as well as the mental health state. These findings are in line with the work of Veenstra et al. who reported that the subdivisions daily emotional interaction, emotional support in problematic situations and social companionship are comparable to a healthy control group [32]. Consistent with previous studies, the majority of our patients presented well-adjusted in terms of social integration [33]. Though Veenstra et al. also reported almost half of the patients had negative effects of the surgery on initiating social or intimate contact, body image [32]. In this context patients after treatment with mega-prosthesis seem to be more satisfied [32, 34]. At least patients in our study had children following the rotationplasty in four cases.

One of our unexpected findings was, that scores for vitality, social functioning and mental health were significantly higher in our series in comparison to a representative German sample [21]. At first glance this finding is hard to understand. However, in comparing our findings to the results of long-term survivors of other malignancies, our study might underline the concept of posttraumatic growth [35, 36]. In this context Sears et al. reported that 83 % of breast cancer patients felt at least one benefit in their disease [36]. These women mentioned that following cancer diagnosis and consecutive therapy they live now more intensively and consciously. Furthermore, women evaluated the social support they received during the illness as a positive aspect. This seems to be an important aspect of coping with the illness, as it has been demonstrated that social support is a significant predictor for a better long-term quality of life in breast cancer patients [36].

Of course functional outcome has a significant impact of on HRQL, as a significant reduction of HRQL was reported in patients with a MSTS <50 % [37]. In our study only one patient with an MSTS of 47 % was found to be below the 50 % threshold. This patient had sustained an Achilles tendon tear with several consecutive revision surgeries. Our other patients presented with an average MSTS of 64 ± 12.41 %. This is in line with the literature reporting MSTS between 63 and 80 % following rotationplasty [38, 39]. Comparative studies showed significantly higher MSTS-scores after endoprosthetic knee replacement than after rotationplasty [39]. Tunn et al. report MSTS-scores of 77 to 87 % after endoprosthetic limb salvage therapy of primary bone tumors in proximal tibia and distal femur, respectively [40]. However, there are also higher rates of postoperative complications reported following endoprosthetic knee replacement. In this context Warrener et al. noted complications in 42 % of patients following arthroplasty and only in 25 % following rotationplasty [39]. Aseptic loosening and mechanical failure are the most common complications [41]. Additionally, rising numbers of periprosthetic infections with multidrug resistant bacteria cause increasing concern [42]. Our patients were highly satisfied with a stable new joint offering high functionality for daily activities. The nature of the operation obviously causes difficulties with complex movements such as heel walking, jumping or walking on uneven terrain [30, 31]. However, most of our patients reported little problems in participating in sports, one of the patients is even a national champion in handicapped swimming. With an average Tegner activity level scale of 4.1 our patients felt able to perform moderately heavy work and some recreational sports. In comparison a healthy cohort achieved nearly two grades more (5.7) and was therefore able to perform heavy labor, competitive sports and recreational sports several times a week. This seems to be in line with the work of Hillmann et al., who reported that 85 % of the patients following rotationplasty were actively participating in “high-level” sports [43].

### Limitations

Several strengths and limitations of the present study have to be considered. On the one hand we focus on an extremely rare surgical entity and enrolled at least 12 patients. The average follow-up of 14 ± 9 years. is, according to the best of our knowledge one of the longest, reported in literature. Furthermore all enrolled patients were clinically seen by a doctor and fulfilled the questionnaires under advice. However, due to the rarity of rotationplasty the small number of patients limits this retrospective study. Moreover, a significant number of patients had deceased due to the disease. This might create a potential bias. Additionally the age at the time of surgery as well as the underlying diagnosis was quite heterogeneous.

## Conclusions

Since limb amputation is associated with lower functional outcome, lower health related quality of life and no positive effects on survival compared to both rotationplasty and endoprosthetic reconstruction, alternative treatment options should be considered first [44–46]. Although recent studies show that modern endoprosthetic reconstructions show equal or even better functional outcomes than rotationplasty, endoprostheses bare the risk of high complication rates [38–42]. The presented long-term results indicate that rotationplasty provides a high HRQL and that patients are satisfied with a good functional outcome regarding activities of daily life. In terms of vitality, social function and mental health our study group showed even significant higher scores compared to a representative German sample in the SF-36v2 survey. In this regard we consider our work to be of distinct relevance regarding the discussion of surgical treatment plans for patients suffering from malignant tumors of the lower thigh and the choice whether to perform a rotationplasty or a endoprosthetic reconstruction.
